# Initiation and completion of treatment for latent tuberculosis infection in migrants globally: a systematic review and meta-analysis

**DOI:** 10.1016/S1473-3099(21)00052-9

**Published:** 2021-12

**Authors:** Kieran Rustage, Jessica Lobe, Sally E Hayward, Kristina L Kristensen, Ioana Margineanu, Ymkje Stienstra, Delia Goletti, Dominik Zenner, Teymur Noori, Manish Pareek, Christina Greenaway, Jon S Friedland, Laura B Nellums, Sally Hargreaves, ESGITM and ESGMYC study groups

**Affiliations:** a The Migrant Health Research Group, Institute for Infection and Immunity, St George's University of London, London, UK; b Department of Internal Medicineand Infectious Diseases, University Medical Center Groningen, University of Groningen, Groningen, Netherlands; c Research Centre for Migration, Ethnicity and Health, University of Copenhagen, Copenhagen, Denmark; d International Reference Laboratory of Mycobacteriology, Statens Serum Institut, Copenhagen, Denmark; e Saint Camillus International University of Health and Medical Sciences, Rome, Italy; f Institute for Population Health Sciences, Queen Mary University of London, London, UK; g European Centre for Disease Prevention and Control, Solna, Sweden; h Department of Respiratory Sciences, University of Leicester, Leicester, UK; i Division of Infectious Diseases, Jewish General Hospital, McGill University, Montreal, QC, Canada; j Center for Clinical Epidemiology, Lady Davis Institute for Medical Research, Jewish General Hospital, McGill University, Montreal, QC, Canada; k Faculty of Medicine and Health Sciences, University of Nottingham, Nottingham, UK; l Faculty of Public Health and Policy, London School of Hygiene & Tropical Medicine, London, UK

## Abstract

**Background:**

Latent tuberculosis infection (LTBI) is one of the most prevalent infections globally and can lead to the development of active tuberculosis disease. In many low-burden countries, LTBI is concentrated within migrant populations often because of a higher disease burden in the migrant's country of origin. National programmes consequently focus on screening and treating LTBI in migrants to prevent future tuberculosis cases; however, how effective these programmes are is unclear. We aimed to assess LTBI treatment initiation and outcomes among migrants, and the factors that influence both.

**Methods:**

For this systematic review and meta-analysis, we searched Embase, MEDLINE, and Global Health, and manually searched grey literature from Jan 1, 2000, to April 21, 2020. We included primary research articles reporting on LTBI treatment initiation or completion, or both, in migrants and excluded articles in which data were not stratified by migrant status, or in which the data were related to outcomes before 2000. There were no geographical or language restrictions. All included studies were quality appraised using recognised tools depending on their design, and we assessed the heterogeneity of analyses using *I*^2^. We extracted data on the numbers of migrants initiating and completing treatment. Our primary outcomes were LTBI treatment initiation and completion in migrants (defined as foreign-born). We used random-effects meta-regression to examine the influence of factors related to these outcomes. The study is registered with PROSPERO (CRD42019140338).

**Findings:**

2199 publications were retrieved screened, after which 39 publications from 13 mostly high-income, low-burden countries were included in our analyses, with treatment initiation and completion data reported for 31 598 migrants positive for LTBI, with not all articles reporting the full pathway from initiation to completion. The pooled estimate for the true proportion of migrants testing positive who initiated treatment was 69% (95% CI 51–84; *I*^2^= 99·62%; 4409 of 8764). The pooled estimate for the true proportion of migrants on treatment in datasets, who subsequently completed it was 74% (95% CI = 66–81; *I*^2^= 99·19%; 15 516 of 25 629). Where data were provided for the entire treatment pathway, the pooled estimate for the true proportion of migrants who initiated and completed treatment after a positive test was only 52% (95% CI 40–64; *I*^2^= 98·90%; 3289 of 6652). Meta-regression showed that LTBI programmes are improving, with more recent reported data (2010–20) associated with better rates of treatment initiation and completion, with multiple complex factors affecting treatment outcomes in migrants.

**Interpretation:**

Although our analysis highlights that LTBI treatment initiation and completion in migrants has improved considerably from 2010–20, there is still room for improvement, with drop out reported along the entire treatment pathway. The delivery of these screening and treatment programmes will require further strengthening if the targets to eradicate tuberculosis in low-incidence countries are to be met, with greater focus needed on engaging migrants more effectively in the clinic and understanding the diverse and unique barriers and facilitators to migrants initiating and completing treatment.

**Funding:**

European Society of Clinical Microbiology and Infectious Diseases, the Rosetrees Trust, the National Institute for Health Research, and the Academy of Medical Sciences.

## Introduction

Approximately 25% of the global population have latent tuberculosis infection (LTBI),^1^, ^2^ and approximately 5–15% of those infected will develop active tuberculosis in their lifetime.^3^, ^4^ Among contacts of patients with pulmonary tuberculosis, the 5-year cumulative risk of LTBI infection is approximately 10%, although this risk appears to be much greater in younger individuals, in whom the 5-year risk was 33·3% in contacts younger than 5 years, and 19·1% in those aged 5–14 years.^5^ Delivering effective LTBI screening and treatment programmes is increasingly emphasised in the control of tuberculosis, with WHO's End TB strategy outlining expanded preventive treatment of people at higher risk of tuberculosis, and encouraging research to improve LTBI detection and treatment.^6^ The first UN high-level meeting on tuberculosis in 2018 set a target to reach 30 million people with preventive treatment for tuberculosis between 2018 and 2022, including 20 million household contacts, and 4 million children younger than 5 years.^7^

**Research in context**

**Evidence before this study**

Reactivation of latent tuberculosis infection (LTBI) is a major driver of tuberculosis incidence worldwide; in countries with a low incidence of tuberculosis, LTBI is disproportionately concentrated among migrants, with national programmes increasingly focusing on the diagnosis and treatment of LTBI in migrants and other high-risk groups. However, little is known about the success of these programmes in engaging migrants and ensuring treatment completion because migrants often face multiple barriers to accessing health care on arrival in the host country. We searched MEDLINE, Embase, and Global Health from Jan 1, 2000, to April 21, 2020 for publications on LTBI treatment initiation and completion in migrants. Before this study we found two reviews on this topic (Sandgren and colleagues, 2016, and Alsdurf and colleagues, 2016); however, neither specifically focused on migrants. Sandgren and colleagues included a small number of studies reporting on migrant outcomes and did not have a formal meta-analysis, and Alsdurf and colleagues used data before 2000 that might not be relevant to current policy. Other studies have reported on migrant-specific outcomes in LTBI programmes globally, but the focus is often on screening practices rather than outcomes and evidence in this area has not yet been effectively consolidated.

**Added value of this study**

This is a comprehensive systematic review and meta-analysis exploring LTBI treatment initiation and completion specifically among migrant populations. We report LTBI treatment outcome data on 31 598 migrants from 2000 onwards in 13 low-incidence countries (<10 cases per 100 000 population). The research provides robust insights into the proportion of individuals initiating and completing treatment, using meta-regression to explore heterogeneity. The data showed that between 2000 and 2020, the pooled estimate for the true proportion of migrants who tested positive for LTBI had initiated treatment was 4409 (69%) of 8764 and of those starting treatment, 15 516 (74%) of 25 629 completed it. Among studies capturing data on both initiation and completion, the pooled estimate for the true proportion of migrants who tested positive for LTBI who successfully initiated and completed treatment was only 3289 (52%) of 6652, with dropouts reported along the entire treatment pathway. The data also indicate higher initiation and completion of treatment between 2010 and 2020 than before 2010 with renewed focus on this approach to tuberculosis control, and a trend toward more positive outcomes among migrants in programmes in the WHO European region. The data showed that multiple complex factors affect treatment outcomes in migrants, including patient demographics and health systems.

**Implications of all the available evidence**

Delivery of LTBI programmes will need to be strengthened to improve outcomes for migrants and meet targets to eradicate tuberculosis in low-incidence countries. Greater focus will need to be placed on engaging migrants more effectively in the clinic, understanding the varied reasons for migrants' declining treatment when testing positive, and ensuring treatment adherence using innovative approaches that are mindful of and sensitive to the unique experiences of this group on arrival to the host country.

In many high-income, low-incidence tuberculosis countries (<10 cases per 100 000 population)^8^ migrants have a greater burden of tuberculosis, and tend to be younger, than native-born populations.^9^, ^10^ For example, 77·3% of first-time asylum seekers in the 27 EU countries in 2019 were younger than 35 years.^11^ LTBI prevalence is probably higher in some migrant populations, with refugee children estimated to have LTBI prevalence rates of around 11%.^12^ High LTBI prevalence could drive greater active tuberculosis incidence within these populations, as most cases are likely to be due to re-activation of LTBI.^13^ One retrospective cohort of 142 314 people estimated a tuberculosis incidence rate of 120 cases per 100 000 person-years among migrants compared with an incidence rate of 4 cases per 100 000 person-years in native-born individuals.^10^ The incidence of tuberculosis among migrants is greatest within the first 5 years of arrival, driven by health-related factors (eg, age and comorbid status) and socioeconomic factors (eg, living conditions).^9^, ^14^ WHO has published recommendations for low-incidence countries to consider systematic LTBI testing and treatment in migrants.^15^ Consequently, the European Centre for Disease Prevention and Control (ECDC) has developed guidance for the programmatic management of LTBI in migrants (among other groups), in line with WHO recommendations.^16^

Increasingly, LTBI and tuberculosis screening programmes in low-incidence tuberculosis countries now include migrants from high-burden tuberculosis countries alongside other high-risk groups,^17^ whom are at greater risk of LTBI re-activation than other groups.^14^, ^18^ 6–9 month courses of isoniazid therapy for LTBI are being increasingly replaced with shorter 3–4 month treatment regimens that often confer increased treatment completion and a reduction in complications and side-effects for patients that might have a positive effect on treatment completion.^15^, ^19^ Although availability and use of these regimens varies by country, they could contribute to improved treatment completion among migrant patients.

Uncertainties exist regarding the effectiveness of LTBI screening and treatment programmes involving migrants. A systematic review on infectious disease screening among migrants to Europe revealed that screening often focuses on LTBI, and suggests that 54% of migrants with a positive LTBI test complete treatment.^20^ Another systematic review and meta-analysis analysing the LTBI care cascade among multiple population groups estimated that only 54·6% of migrants initiate treatment, and 14·3% of all migrants complete treatment.^21^ Risk factors that specifically affect migrant LTBI treatment outcomes include legal status,^21^ patients' mistrust, and uncertainty around legal entitlements regarding eligibility and access to medical care for migrants among patients and staff,^22^ and language and cultural barriers.^21^, ^23^ More generally, logistical barriers to accessing treatment (such as wait times) and side-effects can affect all patient groups.^21^ Furthermore, clinician-recommended treatment can deviate from national guidelines because of clinician perceptions of the risk–benefit balance of treating LTBI.^21^, ^23^ Facilitators to treatment such as culturally sensitive services, patient involvement and ownership in delivering care, and ensuring high-quality service provider management, aid screening and could benefit LTBI treatment.^20^, ^22^ Understanding facilitators and barriers to LTBI treatment initiation and completion, and key stages in the screening and treatment pathway in which migrants are lost to follow-up, is essential for informing where to target interventions to improve health outcomes for migrants.

We therefore did a systematic review and meta-analysis to explore and assess available evidence on the initiation and completion of LTBI treatment among LTBI-positive migrants globally to better understand whether, and where, efforts to improve current practices could be targeted.

## Methods

### Search strategy and selection criteria

We did a systematic review and meta-analysis to identify and synthesise evidence on rates and correlates of treatment initiation once a migrant had tested positive, and the completion of the full recommended course of LTBI treatment post-screening. We defined migrants as any foreign-born individual.

We included publications reporting primary data on LTBI treatment uptake and adherence in migrant populations treated in the receiving country. Uptake was defined as a migrant initiating treatment after a positive test result. Adherence was defined as a migrant completing the course of required treatment after testing positive. There were no geographical or language restrictions regarding the publications included, and we included both adult and paediatric populations. We excluded case reports and case series because of the small number of individuals that these study types include. We also excluded publications with no primary data or discernible study design (eg, comments, editorials, letters, and reviews). We excluded studies with no clearly identifiable migrant population, or in which the definition of a migrant conflicted with our own (eg, defining migrant status on the basis of ancestry or ethnicity). We searched for publications from Jan 1, 2000, to April 21, 2020, excluding those that reported data from before 2000, or that had data after 2000 that could not be disaggregated. We chose 2000 as cutoff to better represent the rapidly evolving field in LTBI treatment in the past two decades, including contemporary treatments and global initiatives.

We searched the databases MEDLINE, Embase, and Global Health from Jan 1, 2000, to April 21, 2020, using a Boolean search strategy with keywords and relevant medical subheadings (MeSH) pertaining to three main themes: migrants, initiation and completion, and LTBI. Search terms were identified by consulting existing literature and experts in these areas. The search strategy and search terms are available in the appendix (pp 2–4) We explored the grey literature by hand-searching conference proceedings and the websites of relevant non-governmental and other organisations (eg, WHO). We identified additional publications through hand searching the bibliographies of publications included in the analysis. We compiled a list of experts in LTBI and emailed them formally requesting grey literature and publications that they were familiar with or programmatic data they were at liberty to divulge.

**Figure 1:**
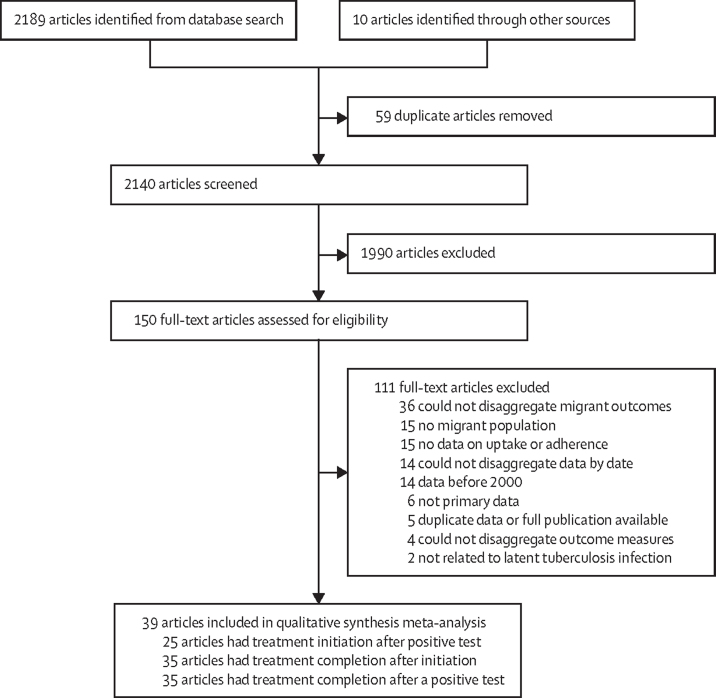
PRISMA flow diagram of the article screening process

Two reviewers duplicated the title and abstract screening and full-text screening (KR, JL), which was done using the web-based application Rayyan.^24^ The reasons for excluding studies during full-text screening are shown in figure 1. Data were extracted by two reviewers (KR and JL). Where there were discrepancies in screening decision, or the extracted data between the primary reviewers, a third reviewer (LBN or SH) mediated screening decisions and ensured the accuracy of extracted data.

This research was done in line with the Preferred Reporting Items for Systematic Reviews and Meta-Analyses (PRISMA),^25^ and registered with PROSPERO (CRD42019140338).

### Data analysis

We used a piloted extraction form, which was developed among the research team, to retrieve data relating to treatment initiation and completion, alongside summary data on the study such as the design, dates, and location. We extracted summaries of analysis relating to positive and negative factors associated with the recorded initiation and completion of treatment within each publication. Where data were available, we categorised each study according to: the WHO region where it took place; the decade the data were related to (2000–09 or 2010–20); the type of migrants being studied (refugee, asylum seeker, undocumented, foreign-born); the treatment regimens used (6 months isoniazid, 9 months isoniazid, 6–9 months isoniazid, 3 months isoniazid plus rifampicin, 4 months rifampicin, mixed [including rifampicin and isoniazid regimens], and uncertain treatment regimen); treatment setting (a single site for screening and treatment, a single site for treatment only, multiple sites for screening and treatment, and multiple sites for treatment only; appendix pp 5–6).

We did all analysis in Stata/SE 16 using the metapreg command to calculate pooled proportions, as well as univariable and multivariable meta-regressions. Freeman-Tukey double arcsine transformation was used, and 95% CIs were calculated using Clopper-Pearson exact CIs in all instances.^26^, ^27^ Heterogeneity between studies was estimated using the *I*^2^ statistic; because of the heterogeneity of the included studies, all analyses used inverse-variance weighted random-effects models using the DerSimonian-Laird method.

We did a meta-analysis to calculate pooled estimates for three primary outcomes: the proportion of migrants positive for LTBI who initiated treatment, the proportion of migrants who, having initiated LTBI treatment, had completed it, and the overall proportion of migrants positive for LTBI who both successfully initiated and completed treatment. We also did a random-effects meta-regression to analyse the influence of the variables indicated a priori and categorised during the extraction process on the three key outcomes. These variables were analysed using univariable meta-regression and those resulting in a p value of less than 0·25 when testing heterogeneity between subgroups (appendix p 7) were included in a multivariable model that controlled for all other co-variates (table).

**Table:** Meta-regression incorporating factors in which univariable analysis p value <0·25

		**Point estimate**	**p value**	**Raw coefficient (95% CI) change in outcome compared with the reference group**
**Treatment initiation among migrants positive for LTBI (univariable analysis only)**				
Time period				
	2000s	85%	(ref)	(ref)
	2010s	43%	<0·0001	2·10 (0·51 to 3·61)
**Treatment completion among migrants that initiate treatment**				
WHO region				
	Region of the Americas	66%	(ref)	(ref)
	European region	87%	<0·0001	0·93 (0·33 to 1·54)
	Western Pacific region	75%	0·82	0·09 (−0·71 to 0·89)
Time period				
	2000s	57%	(ref)	(ref)
	2010s	88%	<0·0001	1·45 (0·82 to 2·10)
Treatment regimen				
	9 months isoniazid	57%	(ref)	(ref)
	6 months isoniazid	91%	0·12	1·17 (−0·31 to 2·65)
	6–9 months isoniazid	71%	0·21	0·09 (−0·75 to 0·93)
	3 months isoniazid plus rifampicin	86%	0·48	−0·37 (−1·39 to 0·66)
	4 months rifampicin	89%	0·020	1·37 (0·24 to 2·50)
	Mixed, including rifampicin and isoniazid	75%	0·59	0·21 (−0·55 to 0·96)
	Unclear	56%	0·22	−0·65 (−1·68 to 0·39)
**Treatment completion in migrants positive for LTBI**				
WHO region				
	Region of the Americas	44%	(ref)	(ref)
	European region	70%	0·020	1·01 (0·19 to 1·83)
	Western Pacific region	27%	0·14	−0·89 (−2·07 to 0·29)
Time period				
	2000s	26%	(ref)	(ref)
	2010s	66%	<0·0001	1·49 (0·56 to 2·42)
Treatment regimen				
	9 months isoniazid	37%	(ref)	(ref)
	6 months isoniazid	42%	0·74	0·35 (−1·76 to 2·47)
	6–9 months isoniazid	50%	0·20	−1·00 (−2·51 to 0·51)
	3 months isoniazid plus rifampicin	74%	0·42	−0·55 (−1·91 to 0·81)
	4 months rifampicin	70%	0·96	0·05 (−1·76 to 1·86)
	Mixed, including rifampicin and isoniazid	50%	0·58	−0·35 (−1·57 to 0·87)
	Unclear	38%	0·070	−1·01 (−2·11 to 0·09)

Results of statistical analysis of LTBI treatment initiation and completion outcomes in migrants. LBTI=latent tuberculosis infection.

Narrative synthesis of positive and negative factors associated with LTBI treatment initiation and completion was done by extracting and reporting on factors found to significantly influence LTBI treatment initiation and completion within the included publications' analyses.

Quality assessment was done for each included article using established appraisal tools (appendix pp 8–9). Cross-sectional studies were assessed using the Joanna Briggs Institute checklist for prevalence studies.^28^ Cohort studies^29^ and randomised controlled trials^30^ were assessed using their respective critical appraisal skills programme checklists. Using these tools, articles were given a quality score (appendix 5–6). For case-series and cohort studies, scores were calculated as a total out of the maximum number of applicable questions (pp 8–9). Quality scores are reported but studies were not excluded on the basis of quality to increase transparency and ensure that all available evidence in this field was reported. Critical appraisal was done in duplicate (KR and SEH).

### Role of the funding source

The funder of the study had no role in study design, data collection, data analysis, data interpretation, or writing of the report.

## Results

Database and grey literature searches yielded 2199 publications, of which the full texts of 150 publications were screened and 39 were included in the review and meta-analysis; (figure 1; appendix 5–6). The 39 included publications contained data on 31 598 migrants positive for LTBI. Studies were done in 13 countries, across three WHO regions (European region, region of the Americas, and Western Pacific region): Australia (n=4 studies),^31^, ^32^, ^33^, ^34^ Canada (n=3),^35^, ^36^, ^37^ Germany (n=1),^38^ Israel (n=1),^39^ Japan (n=1),^40^ the Netherlands (n=2),^41^, ^42^ Norway (n=2),^43^, ^44^ South Korea (n=1),^45^ Spain (n=3),^46^, ^47^, ^48^ Sweden (n=1),^49^ Switzerland (n=1),^50^ UK (n=4),^51^, ^52^, ^53^, ^54^ and USA (n=15).^55^, ^56^, ^57^, ^58^, ^59^, ^60^, ^61^, ^62^, ^63^, ^64^, ^65^, ^66^, ^67^, ^68^, ^69^

**Figure 2:**
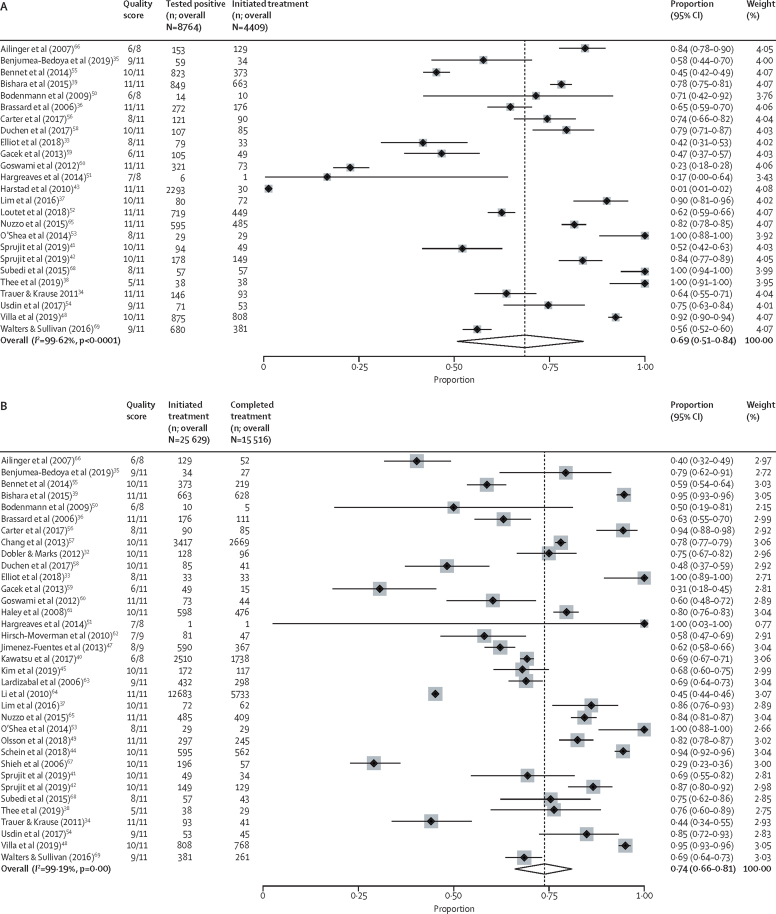

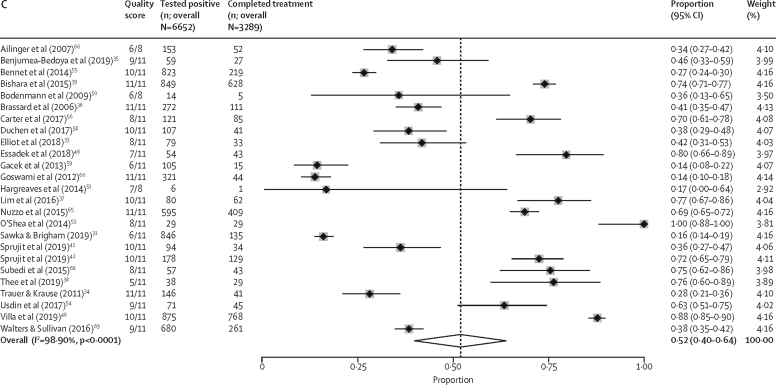
Meta-analysis forest plots estimating migrant LTBI patient treatment initiation and completion Forest plots showing the proportion of migrants positive for LTBI who initiated treatment (A), the proportion of migrants who, having initiated LTBI treatment, had completed it (B), and the proportion of migrants positive for LTBI who successfully initiated and completed treatment (C). LTBI=latent tuberculosis infection.

**Figure 3:**
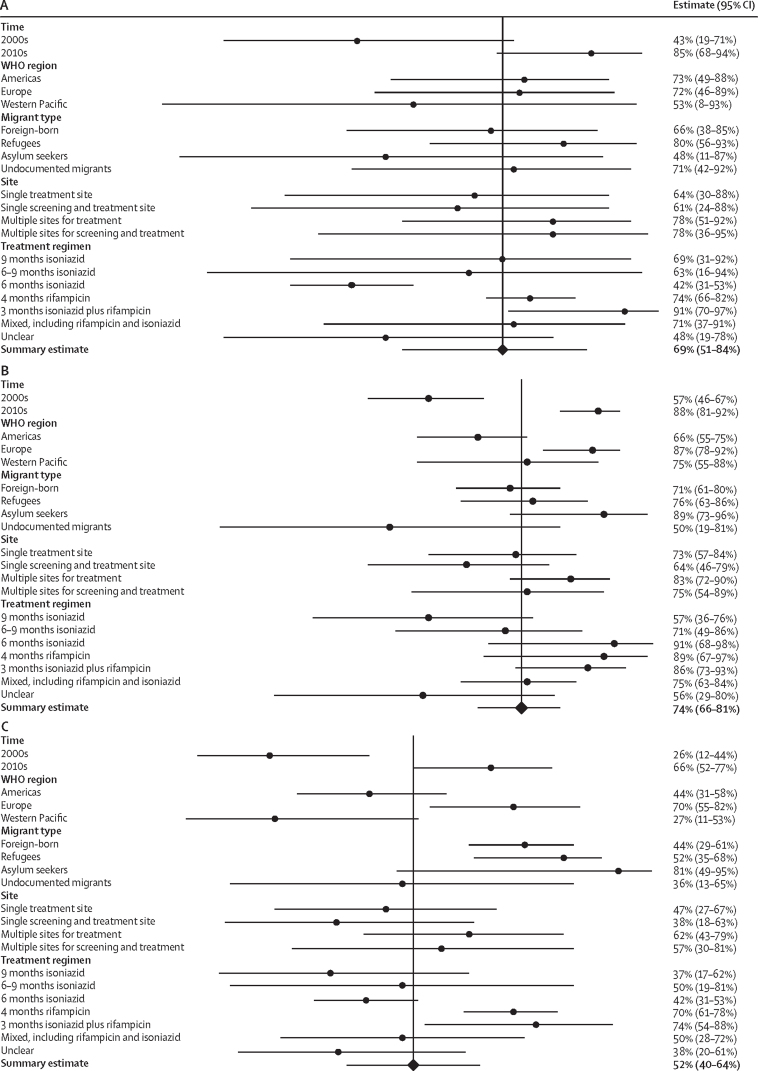
Subgroup forest plots of individual variable estimates (time, WHO region, migrant type, site, and treatment regimen) for migrant LTBI patient treatment initiation and completion Forest plots showing the proportion of migrants positive for LTBI who initiated treatment (A), the proportion of migrants who, having initiated LTBI treatment, had completed it (B), and the proportion of migrants positive for LTBI who successfully initiated and completed treatment (C). LTBI=latent tuberculosis infection.

In total, 25 studies with outcome data on 8764 migrants were used to calculate the proportion of individuals initiating treatment.^33^, ^34^, ^35^, ^36^, ^37^, ^38^, ^39^, ^41^, ^42^, ^43^, ^48^, ^50^, ^51^, ^52^, ^53^, ^54^, ^55^, ^56^, ^58^, ^59^, ^60^, ^65^, ^66^, ^68^, ^69^ The pooled estimate for the true proportion of LTBI treatment initiation in migrants positive for LTBI across the included studies was calculated as 69% (95% CI 51–84; 4409 of 8764, *I*^2^=99·62% (95% CI 99·58–99·66; figure 2A). In univariable meta-regression, only the decade in which individuals enrolled was found to have a significant effect. Specifically, studies that reported data in the 2010s showed higher proportions of treatment initiation (85% [95% CI 68–94]; p<0·0001) than studies from the 2000s (43% [95% CI 19–71]; table; figure 3A).

Demographic and patient factors associated with non-initiation of treatment reported in the studies were being of sub-Saharan African origin^55^ and being a refugee from sub-Saharan Africa, northern Africa, or the Middle East.^69^ Factors positively associated with treatment initiation included having an African birthplace,^60^ close contact with a person with infectious tuberculosis,^60^ and non-employment as a reason for LTBI testing.^60^ One study reported significantly higher treatment initiation in refugees than in other foreign-born groups and the native-born population.^65^ Reported clinical and systems-related factors included testing with Quantiferon Test in refugees, which led to significantly higher proportion of individuals initiating treatment than when using Mantoux tuberculin skin testing.^69^ However, other studies found that non-initiation was associated with treatment not being offered^59^ and that recommendation to treat varied by primary care clinic.^52^

35 studies with outcome data on 25 629 migrants (of whom 15 516 completed treatment) were used to calculate the proportion of individuals completing treatment subsequent to initiation.^32^, ^33^, ^34^, ^35^, ^36^, ^37^, ^38^, ^39^, ^40^, ^41^, ^42^, ^44^, ^45^, ^47^, ^48^, ^49^, ^50^, ^51^, ^53^, ^54^, ^55^, ^56^, ^57^, ^58^, ^59^, ^60^, ^61^, ^62^, ^63^, ^64^, ^65^, ^66^, ^67^, ^68^, ^69^ The pooled estimate for the true proportion of LTBI treatment completion among migrants initiating treatment was 74% (95% CI 66–81; 15 516 of 25 629; *I*^2^=99·19% [95% CI 99·09–99·28]; figure 2B). In univariable meta-regression WHO region, the time period the data were related to, and the treatment regimen used were all significantly associated with treatment completion. In the multivariable model, migrants were more likely to complete treatment in the 2010s (88% [95% CI 81–92]; p<0·0001) than in the 2000s (57% [95% CI 46–67]; ref). Migrants treated in the WHO European region were significantly more likely to complete treatment (87% [95% CI 78–92]; p<0·0001) than migrants in the region of the Americas (66% [95% CI 55–75]; ref) or the Western Pacific region (75% [95% CI 55–88]; p=0·82). Analysing by treatment regimen, migrants treated with 4 months of rifampicin were significantly more likely to complete treatment (89% [95% CI 67–97]; p=0·020) than migrants prescribed 9 months of isoniazid (ref); no other treatment regimen showed significant differences (table; figure 3B).

Demographic and patient-related factors associated with non-completion include unemployment,^47^ education level,^47^ and lack of family support.^47^ Demographic and patient-related factors associated with treatment completion included African or Asian birthplace (compared with a birthplace in the USA, Canada, western Europe, or Japan),^60^ being a refugee from South Asia,^69^ foreign-birth,^44^, ^64^ having resided in the host country for less than 5 years,^63^ refugee status^65^ as well as migration from Europe or Asia.^57^ Two studies found no association between any demographic factors and treatment completion in either direction.^66^, ^67^ Clinical and system-related factors associated with non-completion included side-effects,^32^, ^39^, ^66^ being prescribed a 3-month isoniazid plus rifampicin regimen compared with 6 months of isoniazid,^47^ receiving 4 months of rifampicin compared with 9 months of isoniazid,^63^ 6 months of isoniazid versus 9 months of isoniazid,^49^ receiving LTBI treatment in combination with immunosuppressive treatment,^49^ receiving treatment more recently (after 2013),^49^ daily and weekly directly observed therapy versus self-administered care,^44^ and rifampicin versus isoniazid treatment.^69^

25 studies containing outcome data on 6652 migrants (of whom 3289 had initiated and completed treatment) were used to calculate the overall proportion of migrants positive for LTBI who completed treatment.^31^, ^33^, ^34^, ^35^, ^36^, ^37^, ^38^, ^39^, ^41^, ^42^, ^46^, ^48^, ^50^, ^51^, ^53^, ^54^, ^55^, ^56^, ^58^, ^59^, ^60^, ^65^, ^66^, ^68^, ^69^ The pooled estimate for the true proportion of migrants completing treatment after screening positive for LTBI was 52% (95% CI 40–64; 3289 of 6652; *I*^2^=98·90% [95% CI 98·73–99·06]; figure 2C). In univariable meta-regression, WHO region and time period of treatment receipt were significantly associated with treatment completion. In the multivariable model, migrants were more likely to complete treatment in the 2010s (66% [95% CI 52–77]; p<0·0001) than migrants in the 2000s (26% [95% CI 12–44]; ref). Migrants treated in the WHO European region were also significantly more likely to complete treatment (70% [95% CI 55–82]; p=0·020) than migrants in the region of the Americas (44% [95% CI 31–58]; ref) and the Western Pacific region (27% [95% CI 11–53]; p=0·14). In our analysis, no other factors were found to be significantly associated with completion (table; figure 3C).

## Discussion

This systematic review and meta-analysis comprehensively reports on pathway results for LTBI treatment outcomes for migrants. We identified evidence from 13 mostly high-income, low-incidence tuberculosis countries and regional variation in treatment outcomes in migrants and approaches taken for treatment. Our analysis of 31 598 migrants positive for LTBI within the 39 included studies showed that overall, between 2000 and 2020 in studies reporting on the full initiation and treatment pathway, only 52% of migrants who tested positive for LTBI both initiated and completed treatment. We found that 69% of migrants with a positive LTBI test initiated treatment, and 74% of those who initiated treatment completed it. Overall, these data suggest that there is drop-out along the treatment pathway. These studies highlighted complex barriers and facilitators related to patient demographics and health systems and clinical decision making that affected outcomes. Improvements in the LTBI care cascade are needed, particularly with respect to the initiation of treatment of individuals who test positive. Our analysis suggests that the current approach requires renewed emphasis and strengthening, despite substantial improvements in outcomes in the past decade.

A previous review of LTBI initiation and completion found that treatment initiation rates in migrants ranged between 23% and 97%, and completion rates between 7% and 86%.^70^ A further systematic review and meta-analysis showed treatment initiation of 54·6% and completion of 14·3% in migrants.^21^ Our study builds on these estimates by calculating a pooled rate of initiation and completion specifically for migrant populations. Our estimation that approximately 74% of migrants who initiated LTBI treatment completed it in the period under study is markedly higher and more optimistic than the 14·3% posited by previous research.^21^ However, our data on the entire pathway, from a migrant testing positive through to subsequent initiation and then completion of treatment, showed that our calculated rate of 52% is comparable to other studies, which suggests drop-out along the entire treatment trajectory.

Important sociodemographic and cultural factors might influence the decision of migrant patients to initiate and complete treatment. Stigma and misconceptions about the cause of tuberculosis can effect outcomes, and LTBI diagnosis can be misinterpreted as a tuberculosis diagnosis, with fears of stigma and social impact reflecting those of active tuberculosis.^71^ Clinicians also report difficulties in effectively communicating about LTBI with migrant patients and communities.^23^ Clinical practice and guidance are also important in supporting migrants to initiate treatment whereby differing clinical practices might result in non-initiation because of factors beyond the patients' control.^52^, ^59^, ^69^ Deviation from national guidelines in health care has been evidenced in other research—eg, clinicians thinking that particular migrants are not eligible for preventive treatment, discrepancies around age thresholds for treatment, and other exclusion criteria.^23^, ^72^ ECDC guidelines call for migrants who arrived within the past 5 years from high-tuberculosis-incidence countries to be screened for LTBI using a tuberculin skin test or an interferon-γ release assay and for these individuals to be linked to care and treatment.^73^ Toolkits supporting best practice for such guidelines exist, and could be further adapted to support treatment.^74^

More research is needed to assess facilitators to improve outcomes for LTBI in migrants. Crucially this research must better delineate what it is about being a migrant, or their experiences, that affects outcomes, which could be achieved through increased involvement of migrant communities across the entire research process, from inception to dissemination.

We found conflicting evidence as to whether foreign-born status acts as a barrier or facilitator of treatment initiation and completion in the included studies. Studies using a binary foreign-born status appear to be most ambiguous, with some stating a positive association,^44^, ^64^ and some a negative association.^40^, ^61^ Studies with greater granularity (eg, regional or country-specific analyses) produce a more nuanced picture, with divergent risk profiles for initiation and completion between migrant groups. These findings suggests that factors such as an individual's country of origin, years of residency, and legal status provide important context beyond the scope of foreign-born status alone. Future research taking account of these factors could identify groups requiring greater treatment support. The composition of migrant groups might help to explain the disparate initiation and completion estimates in WHO regions, which have different migration flows.^75^ Interaction between factors associated with a migrants' origin (cultural practices, medical beliefs) and the environment into which they migrate (access to care, availability of tailored services, resultant socioeconomic status), if disentangled, could also explain the differing levels of treatment initiation and completion seen between WHO regions.

Greater granularity in data could also contribute positively toward understanding the cost-effectiveness of LTBI screening and treatment, which continues to be a key question. Previous research indicates that the effectiveness and cost-effectiveness of LTBI screening and treatment is limited by factors such as the large pool of migrants with LTBI, diagnostics, and an insufficiently robust care cascade.^76^ Such programmes are therefore likely to be most effective when targeted, with success and evidence of cost-effectiveness being shown when focusing on migrant children, as an example.^76^, ^77^

Shorter treatment regimens were often associated with better outcomes in the analyses of included studies and might reduce drop-out. Evidence shows that shorter regimens might be as efficacious as longer treatment regimens, and are associated with greater compliance and less discontinuation because of hepatotoxicity and other side-effects.^15^ Shorter regimens are now also more widely recommended.^15^, ^19^ A trial of tuberculosis patients with HIV infections showed that 1 month of daily rifapentine plus isoniazid treatment was non-inferior to 9 months of isoniazid alone, and resulted in significantly more patients completing treatment.^78^ If similar ultra-short course regimens can be prescribed, and other barriers overcome, treatment completion might increase. Although our analyses found little evidence of a significant association between treatment regimen and outcome, the type of regimen appeared to be associated with the time period under study with shorter and rifampicin-containing regimens seen in more recent studies. The influence of treatment regimen should not be discounted because of our analysis and an individual-level patient data meta-analysis is probably better for analysing this association.

The work adheres to PRISMA standards and used a global multi-database search, with the input of expert groups, ensuring as much of the available literature as possible was captured. Furthermore, the meta-analysis provides a robust estimation of global LTBI treatment initiation and completion, and the factors driving these figures were explored through both meta-regression and narrative synthesis.

Our review is limited by the fact that potentially important factors outside of the immediate care setting are not effectively captured by studies in this field at present, yet these factors are likely to influence both LTBI treatment initiation and completion. Furthermore, the binary definition that anyone foreign-born is a migrant can mask important differences resulting from differing legal status, and further excludes first-generation migrants (and beyond) who might have similar barriers. The use of meta-regression is also not without limitations, often being underpowered to detect anything but large associations and any interpretation of the results are susceptible to ecological fallacy. As such, we would consider our analyses relating to meta-regression to be exploratory, and indicative of factors that might warrant more robust investigation in the future. Nevertheless, this systematic review and meta-analysis provides important insight into the treatment outcomes of migrants with LTBI.

## Data sharing

Materials including detailed critical appraisal and characteristics of each study are available in the appendices. The extracted data used within our analysis is available from the St George's University data repository at https://doi.org/10.24376/rd.sgul.13521764.v1.

## Declaration of interests

SH is a freelance Senior Editor for *The Lancet Infectious Diseases* and other *Lancet* journals. All other authors declare no competing interests.
