# Supplementary material for: Initiation and completion of treatment for latent tuberculosis infection in migrants globally: a systematic review and meta-analysis
Source: Lancet Infect Dis. 2021 Dec;21(12):1701–12. doi: 10.1016/S1473-3099(21)00052-9 (PMC8612939; doi:10.1016/S1473-3099(21)00052-9)
Supplement: Supplementary appendix [file mmc1.pdf]

THE LANCET
Infectious Diseases

Supplementary appendix

This appendix formed part of the original submission and has been peer reviewed. We post it as supplied by the authors.

Supplement to: Rustage K, Lobe J, Hayward SE, et al. Initiation and completion of treatment for latent tuberculosis infection in migrants globally: a systematic review and meta-analysis. *Lancet Infect Dis* 2021; published online August 4. https://doi.org/10.1016/S1473-3099(21)00052-9

Initiation and completion of treatment for latent tuberculosis infection in migrants globally: A systematic review and meta-regression analysis

Kieran Rustage,[a] Jessica Lobe,[a] Sally E. Hayward,[a] Kristina L Kristensen,[c] Ioana Margineanu,[b] Ymkje Stienstra,[b] Delia Goletti,[d] Dominik Zenner,[e] Teymur Noori,[f] Manish Pareek,[g] Christina Greenaway,[h] Jon S Friedland,[a] Laura B Nellums,[I] Sally Hargreaves,[a] on behalf of the ESGITM and ESGMYC Study Groups*

Corresponding Author: Sally Hargreaves s.hargreaves@sgul.ac.uk

[a] The Migrant Health Research Group, Institute for Infection and Immunity, St George's University of London, United Kingdom

[b] University of Groningen, University Medical Center Groningen, Department of Internal Medicine/Infectious Diseases, Groningen, The Netherlands

[c] Research Centre for Migration, Ethnicity and Health, University of Copenhagen, Denmark, and International Reference Laboratory of Mycobacteriology, Statens Serum Institut, Denmark

[d] Saint Camillus International University of Health and Medical Sciences, Rome

[e] Institute for Population Health Sciences, Queen Mary University of London, United Kingdom

[f] European Centre for Disease Prevention and Control, Sweden

[g] Department of Respiratory Sciences, University of Leicester, United Kingdom

[h] Division of Infectious Diseases, Jewish General Hospital, McGill University
Center for Clinical Epidemiology, Lady Davis Institute for Medical Research, Jewish General Hospital (CG)

[I] Faculty of Medicine & Health Sciences, Nottingham University

*ESGITM is the European Society of Clinical Microbiology and Infectious diseases Study Group for Infections in Travellers and Migrants; ESGMYC is the European Society of Clinical Microbiology and Infectious diseases Study Group for Mycobacterial Infections.

Appendix 1. Search strategy (with subject headings (/))

Embase Database

1. migrant/ or migrant worker/ or Migrant*.mp.
2. Migrat*.mp.
3.refugee/ or refugee*.mp.
4.asylum seeker/ or asylum seeker*.mp.
5.foreigner*.mp. or foreign worker/
6.foreign born.mp.
7.immigrant/ or immigra*.mp.
8. Emigrants/ or emigrant/ or emigration/ or emigra*.mp.
9. oversea*.mp.
10. foreign student*.mp. or foreign student/
11. international student*.mp.
12. traffick*.mp.
13. 1 or 2 or 3 or 4 or 5 or 6 or 7 or 8 or 9 or 10 or 11 or 12
14. adher*.mp.
15. complian*.mp.
16. deafult.mp.
17. concordan*.mp.
18. treatment outcome/ or treatment outcome*.mp.
19. non adher*.mp.
20. non complian*.mp. or patient compliance/
21. treatment uptake.mp.
22. treatment start.mp.
23. treatment initiation.mp.
24. drop out.mp.
25. follow up/ or loss to follow-up.mp.
26. treatment deferral.mp.
27. treatment completion.mp.
28. treatment success.mp. or treatment outcome/
29. 14 or 15 or 16 or 17 or 18 or 19 or 20 or 21 or 22 or 23 or 24 or 25 or 26 or 27 or 28
30. latent tuberculosis/ or Latent tuberculosis infection.mp.
31. latent TB infection.mp.
32. LTBI.mp.
33. latent tuberculosis.mp.
34. latent TB.mp.
35. latent mycobacterium tuberculosis.mp.
36. inactive tuberculosis infection.mp.
37. inactive tuberculosis.mp.
38.inactive TB.mp.
39. inactive mycobacterium tuberculosis.mp
40. (prophyla* adj3 tuberculosis).mp. [mp=title, abstract, heading word, drug trade name, original title, device manufacturer, drug manufacturer, device trade name, keyword, floating subheading word, candidate term word]
41. 30 or 31 or 32 or 33 or 34 or 35 or 36 or 37 or 38 or 39 or 40
42. 13 and 29 and 41

MEDLINE Database

1. Migrant*.mp. or "Emigration and Immigration"/
2. migrat*.mp. [mp=title, abstract, original title, name of substance word, subject heading word, floating sub-heading word, keyword heading word, organism supplementary concept word, protocol supplementary concept word, rare disease supplementary concept word, unique identifier, synonyms]
3. "Emigrants and Immigrants"/ or Refugees/ or refugee*.mp.
4. asylum seeker*.mp.
5. foreigner*.mp.
6. foreign born.mp.
7. non-native*.mp.
8. immigra*.mp.
9. emigra*.mp.
10. oversea*.mp.
11. foreign student*.mp.
12. International Student*.mp.
13. traffick*.mp.
14. 1 or 2 or 3 or 4 or 5 or 6 or 7 or 8 or 9 or 10 or 11 or 12 or 13
15. adher*.mp.
16. complian*.mp.
17. default.mp.
18. concordan*.mp. [mp=title, abstract, original title, name of substance word, subject heading word, floating sub-heading word, keyword heading word, organism supplementary concept word, protocol supplementary concept word, rare disease supplementary concept word, unique identifier, synonyms]
19. Treatment Outcome/ or treatment outcome*.mp.
20. non-adher*.mp. [mp=title, abstract, original title, name of substance word, subject heading word, floating sub-heading word, keyword heading word, organism supplementary concept word, protocol supplementary concept word, rare disease supplementary concept word, unique identifier, synonyms]
21. non-complian*.mp. [mp=title, abstract, original title, name of substance word, subject heading word, floating sub-heading word, keyword heading word, organism supplementary concept word, protocol supplementary concept word, rare disease supplementary concept word, unique identifier, synonyms]
22. Treatment uptake.mp.
23. treatment start.mp.
24. treatment initiation.mp.
25. drop out.mp.
26. loss to follow-up.mp.
27. treatment deferral.mp.
28. treatment completion.mp.
29. treatment success.mp.
30. 15 or 16 or 17 or 18 or 19 or 20 or 21 or 22 or 23 or 24 or 25 or 26 or 27 or 28 or 29
31. Latent tuberculosis infection.mp. or Latent Tuberculosis/
32. Latent TB infection.mp.
33. LTBI.mp.
34. latent TB.mp.
35. latent Mycobacterium tuberculosis.mp. [mp=title, abstract, original title, name of substance word, subject heading word, floating sub-heading word, keyword heading word, organism supplementary concept word, protocol supplementary concept word, rare disease supplementary concept word, unique identifier, synonyms]
36. inactive tuberculosis infection.mp.
37. inactive TB infection.mp.
38. Inactive Tuberculosis.mp.
39. Inactive TB.mp.
40. Inactive Mycobacterium Tuberculosis.mp.
41. (prophyla* adj3 tuberculosis).mp. [mp=title, abstract, original title, name of substance word, subject heading word, floating sub-heading word, keyword heading word, organism supplementary concept word, protocol supplementary concept word, rare disease supplementary concept word, unique identifier, synonyms]
42. 31 or 32 or 33 or 34 or 35 or 36 or 37 or 38 or 39 or 40 or 41
43. 14 and 30 and 42

Global Health Database

1. migrant labour/ or migrant farm workers/ or Migrant*.mp.
2. Migrat*.mp. [mp=abstract, title, original title, broad terms, heading words, identifiers, cabicodes]
3. Refugee*.mp. or refugees/
4. asylum seeker*.mp.
5. foreigner*.mp.
6. foreign-born.mp.
7. non-native*.mp.
8. immigra*.mp.
9. emigra*.mp. [mp=abstract, title, original title, broad terms, heading words, identifiers, cabicodes]
10. Oversea*.mp.
11. foreign student*.mp. or foreign students.sh.
12. International Student*.mp.
13. traffick*.mp.
14. 1 or 2 or 3 or 4 or 5 or 6 or 7 or 8 or 9 or 10 or 11 or 12 or 13
15. Adher*.mp.
16. complian*.mp. [mp=abstract, title, original title, broad terms, heading words, identifiers, cabicodes]
17. default.mp.
18. concordan*.mp. [mp=abstract, title, original title, broad terms, heading words, identifiers, cabicodes]
19. treatment outcome*.mp.
20. non-adher*.mp. or patient compliance.sh.
21. non-complian*.mp. [mp=abstract, title, original title, broad terms, heading words, identifiers, cabicodes]
22. treatment uptake.mp.
23. treatment start.mp.
24. treatment initiation.mp.
25. drop out.mp.
26. loss to follow-up.mp.
27. treatment deferral.mp.
28. treatment completion.mp.
29. treatment success.mp. or treatment failure.sh.
30. 15 or 16 or 17 or 18 or 19 or 20 or 21 or 22 or 23 or 24 or 25 or 26 or 27 or 28 or 29
31. Latent Tuberculosis Infection.mp.
32. latent tb infection.mp.
33. LTBI.mp.
34. latent tuberculosis.mp.
35. latent TB.mp.
36. latent mycobacterium tuberculosis.mp.
37. inactive tuberculosis infection.mp.
38. inactive tb infection.mp.
39. inactive tuberculosis.mp.
40. inactive tb.mp.
41. inactive mycobacterium tuberculosis.mp.
42. (prophyla* adj3 tuberculosis).mp. [mp=abstract, title, original title, broad terms, heading words, identifiers, cabicodes]
43. 31 or 32 or 33 or 34 or 35 or 36 or 37 or 38 or 39 or 40 or 41 or 42
44. 14 and 30 and 43

Appendix 2. Characteristics of included studies

INH = Isoniazid, RMP = Rifampicin, N.R = Not Reported

Citation	Location	StudyType	Study Quality	Dates	Average Age of migrant Population Reported	LTBI diagnostic used	Treatment Adherence definition	LTBI positive migrants (N)	Migrants initiating treatment (n)	Migrants completing treatment (n)	WHO region assigned	Time period Assigned	Migrant Type Assigned	Treatment Regimen assigned
Ailinger, et al.[62] *2007*	United States	Cross-sectional	6/8	2004-2005	Mean age of 26.1 (SD = 6.6)	N.R	Completion of 270 doses in 12 months	153	129	52	Americas	2000s	Foreign-born	9 Month INH
Benjumea-Bedoya, et al.[31] *2019*	Canada	Retrospective cohort	9/11	Jan 2015 – Dec 2015	Mean age of 32.5 (SD =8.9)	IGRA	≥80% of INH doses within 12 months	59	34	27	Americas	2010s	Refugees	9 Month INH
Bennet, et al.[51] *2014*	United States	Retrospective cohort	10/11	Jan 2010-Oct 2012	Median age of 31	TST, IGRA	N.R	823	373	219	Americas	2010s	Refugees	6-9 Month INH
Bishara, et al.[35] *2015*	Israel	Retrospective cohort	11/11	2005-2011	Mean age of 28.2 (SD = 16.9)	TST	26 supervised doses with 9 months	849	663	628	Europe	.	Foreign-born	6-9 Month INH
Bodenmann, et al.[46] *2009*	Switzerland	Cross-sectional	6/8	Jan 2007 – Jul 2007	Mean age of 34.8	IGRA	N.R	14	10	5	Europe	2000s	Undocumented Immigrants	Unclear
Brassard, et al.[32] *2006*	Canada	Retrospective cohort	11/11	1998 - 2003	unclear	TST	>80% prescribed doses taken with 43 weeks of initiating therapy	507	176	111	Americas	2000s	Foreign-born	9 Month INH
Carter, et al.[52] *2017*	United States	Retrospective cohort	8/11	Aug 2012-Apr 2016	Median age of 31.8 (Range = 18 – 70)	IGRA	120 doses within 6 months	121	90	85	Americas	2010s	Refugees	4 Month RMP
Chang, et al.[53] *2013*	United States	Retrospective cohort	10/11	Jan 2000-Dec 2002	Median age of home follow-up of 22 (interquartile range = 10-31); Median age of clinic follow-up of 24 (interquartile range = 13-35)	TST	≥6 months of INH within 9 months	3417	3417	2669	Americas	2000s	Foreign-born	6-9 Month INH
Dobler, Marks.[28] *2012*	Australia	Retrospective Cohort	10/11	Jan 2000-Dec 2010	Mean age of 27 (SD = 16)	TST	6 monthly bottles of INH collected by patient	128	128	96	Western Pacific	.	Foreign-born	6 Month INH
Duchen, et al.[54] *2017*	United States	Retrospective cohort	11/11	Jan 2009-Apr 2012	Mean age of 35.2 (SD = 11.3)	TST	>90% of doses received & missed less than three daily doses per month	107	85	41	Americas	.	Refugees	Mixed (inc. RMP & INH regimens)
Elliot, et al.[29] *2018*	Australia	Retrospective cohort	8/11	2007 - 2010	Unclear	TST, IGRA	6 Months of preventive therapy	79	33	33	Western Pacific	.	Refugees	6 Month INH
Essadek, et al.[42] *2018*	Spain	Retrospective cohort	7/11	2012-2014	unclear	N.R	N.R	54	n/a	43	Europe	2010s	Foreign-born	Unclear
Gacek, et al.[55] *2013*	United States	Retrospective cohort	6/11	Jan 2007-Dec 2008	N.R	TST	N.R	105	49	15	Americas	2000s	Foreign-born	Unclear
Goswami, et al.[56] *2012*	United States	Prospective cohort	11/11	Jan 2008-May 2009	Unclear	TST	N.R	321	73	44	Americas	2000s	Foreign-born	Mixed (inc. RMP & INH regimens)
Haley, et al.[57] *2008*	United States	Retrospective cohort	10/11	Feb 2000-Feb 2004	unclear	TST	4 pill bottles provided & provider determination treatment was complete	598	598	476	Americas	2000s	Foreign-born	4 Month RMP
Hargreaves, et al.[47] *2014*	United Kingdom	Cross-sectional	7/8	2013	unclear	IGRA	N.R	6	1	1	Europe	2010s	Foreign-born	Unclear
Harstad, et al.[39] *2010*	Norway	Retrospective cohort	11/11	Jan 2005-Jun 2006	N.R	TST	N.R	2293	30	n/a	Europe	2000s	Asylum Seekers	Unclear
Hirsch-Moverman, et al.[58] *2010*	United States	RCT	7/9	2002-2005	N.R	N.R (CDC guidelines)	According to CDC guidelines	81	81	47	Americas	2000s	Foreign-born	9 Month INH
Jimenez-Fuentes, et al.[43] *2013*	Spain	RCT	8/9	Apr 2001-Apr 2005	26.1	TST	>80% of the prescribed dose taken at each follow-up visit & attendance at clinics	590	590	367	Europe	2000s	Foreign-born	Mixed (inc. RMP & INH regimens)
Kawatsu, et al.[36] *2017*	Japan	Cross-sectional	6/8	2007 - 2014	unclear	N.R	≥180 days treatment duration & recorded as treatment completed	2510	2510	1738	Western Pacific	.	Foreign-born	6-9 Month INH
Kim, et al.[41] *2019*	South Korea	Prospective cohort	10/11	2008	Mean age of 35.4 (SD = 10)	TST, IGRA	Finishing ≥80% of prescribed pills	172	172	117	Western Pacific	2000s	Refugees	Mixed (inc. RMP & INH regimens)
Lardizabal, et al.[59] *2006*	United States	Retrospective cohort	9/11	2000-2003	unclear	N.R	Pill count & extensive discussion between nurses and case managers	432	432	298	Americas	2000s	Foreign-born	Mixed (inc. RMP & INH regimens)
Li, et al.[60] *2010*	United States	Retrospective cohort	11/11	Jan 2002-Aug 2004	unclear	TST	Attendance at follow-up appointments	12683	12683	5733	Americas	2000s	Foreign-born	Mixed (inc. RMP & INH regimens)
Lim, et al.[33] *2016*	Canada	Retrospective cohort	10/11	2014 - 2016	unclear	TST, IGRA	Filling at least 75% of treatment doses	80	72	62	Americas	2010s	Refugees	3 Month INH + RMP
Loutet, et al.[48] *2018*	United Kingdom	Retrospective cohort	11/11	Aug 2014-Aug 2015	unclear	IGRA	N.R	719	449	n/a	Europe	2010s	Foreign-born	3 Month INH + RMP
Nuzzo, et al.[61] *2015*	United States	Retrospective cohort	11/11	Feb 2009-Mar 2011	unclear	TST, IGRA	≥6 months INH and attendance at final appointment, or ≥3 months RIF and attendance at final appointment	595	485	409	Americas	.	Foreign-born	Mixed (inc. RMP & INH regimens)
O'Shea, et al.[49] *2014*	United Kingdom	Prospective cohort	8/11	Feb 2012 – Sep 2012	Range = 18-21	TST, IGRA	N.R	29	29	29	Europe	2010s	Foreign-born	3 Month INH + RMP

Olsson, et al.[45] 2018	Sweden	Retrospective cohort	11/11	Jan 2008-Mar 2016	N.R	TST	Collected the prescribed treatment	297	297	245	Europe	.	Asylum Seekers	Mixed (inc. RMP & INH regimens)
Sawka, Brigham.[27] 2019	Australia	Retrospective cohort	6/11	Jan 2013-Dec 2017				846	n/a	135	Western Pacific	2010s	Foreign-born	Unclear
Schein, et al.[40] 2018	Norway	Prospective cohort	10/11	2016	Unclear	IGRA	As reported by responsible clinician & verified by duration of treatment	595	595	562	Europe	2010s	Foreign-born	Mixed (inc. RMP & INH regimens)
Shieh, et al.[63] 2006	United States	Prospective cohort	10/11	Jul 2002-Sep 2003	unclear	TST	N.R	196	196	57	Americas	2000s	Foreign-born	6-9 Month INH
Sprujit, Erkens, et al.[37] 2019	Netherlands	Prospective Cohort	10/11	Mar 2016 – Sep 2016	Unclear	TST, IGRA	N.R	94	49	34	Europe	.	Foreign-born	3 Month INH + RMP
Sprujit, Haile, et al.[38] 2019	Netherlands	Prospective cohort	10/11	Nov 2016-Dec 2017	Unclear	IGRA	National Guidelines	178	149	129	Europe	2010s	Asylum Seekers	3 Month INH + RMP
Subedi, et al.[64] 2015	United States	Retrospective cohort	8/11	2010-2012	N.R	TST, IGRA	N.R	57	57	43	Americas	2010s	Refugees	Unclear
Thee, et al.[34] 2019	Germany	Prospective cohort	5/11	2016	Median age of 16.8 (interquartile range = 16-17.2)	IGRA	N.R	38	38	29	Europe	2010s	Refugees	3 Month INH + RMP
Trauer, Krause.[30] 2011	Australia	Prospective cohort	11/11	Feb 2006-Jan 2009	Median age of 15 (Interquartile range = 7-29)	TST	≥80% of 9-month doses taken within 12 months	146	93	41	Western Pacific	2000s	Refugees	9 Month INH
Usdin, et al.[50] 2017	United Kingdom	Prospective cohort	9/11	Feb 2014 – Mar 2014	N.R	IGRA	N.R	71	53	45	Europe	2010s	Foreign-born	3 Month INH + RMP
Villa, et al.[44] 2019	Spain	Prospective Cohort	10/11	Jan 2016-Dec 2017	unclear	TST, IGRA	Patient self-reporting	875	808	768	Europe	2010s	Asylum Seekers	Mixed (inc. RMP & INH regimens)
Walters, Sullivan.[65] 2016	United States	Retrospective cohort	9/11	Nov 2009-Apr 2011	23.8 (SD = 11.9)	TST, IGRA	N.R	680	381	261	Americas	.	Refugees	Mixed (inc. RMP & INH regimens)

Appendix 3. Univariable meta-regression analysing heterogeneity between sub-groups

**Analyses**	**Results**	
..	**Variable**	**P-value**
**Treatment initiation amongst LTBI positive Migrants**	**..**	**..**
..	**Region**	0.82
..	**Time period**	0.05
..	**Screening & Treatment Setting**	0.81
..	**Migrant Type**	0.76
..	**Treatment Regimen**	0.44
**Treatment completion amongst migrants that initiate treatment**	..	..
..	**Region**	0.01
..	**Time period**	0.00
..	**Screening & Treatment Setting**	0.26
..	**Migrant Type**	0.35
..	**Treatment Regimen**	0.12
**Treatment completion amongst LTBI positive migrants**	..	..
..	**Region**	0.02
..	**Time period**	0.01
..	**Screening & Treatment Setting**	0.47
..	**Migrant Type**	0.37
..	**Treatment Regimen**	0.28

Appendix 4. Results of critical appraisal carried out on included studies. Quality scores were calculated as the cumulative numbers of "Yes" responses to the appraisal questions.

Cross-Sectional (prevalence) Studies	***Citation***	***Study Type***	***Score***	1. Were criteria for inclusion in the sample clearly defined?	2. were the study subjects and the setting described in detail?	3. Was the exposure measured in a valid and reliable way?	4. Were objective, standard criteria used for measurement of the condition?	5. Were confounding factors identified?	6. Were strategies to deal with confounding factors stated?	7. Were the outcomes measured in a valid and reliable way?	8. Was appropriate statistical analysis used?			
	Ailinger, et al. 2007	Cross-sectional	6/8	Yes	Yes	Unclear	No	Yes	Yes	Yes	Yes			
	Bodenmann, et al. 2009	Cross-sectional	6/8	yes	yes	Yes	Yes	Yes	No	Yes	no			
	Hargreaves, et al. 2014	Cross-sectional	7/8	Yes	Yes	Yes	Yes	Yes	No	Yes	Yes			
	Kawatsu, et al. 2017	Cross-sectional	6/8	Yes	Yes	Unclear	Unclear	Yes	Yes	Yes	Yes			
Cohort Studies (Prospective and Retrospective)	***Citation***	***Study Type***	***Score***	1. Did the study address a clearly focused issue?	2. Was the cohort recruited in an acceptable way?	3. Was the exposure accurately measured to minimise bias?	4. Was the outcome measure accurately measured to minimise bias?	5a. Have the authors identified all important confounding factors?	5b. Have they taken account of the confounding factors in the design and/or analysis?	6a. Was the follow up of subjects complete enough?	6b. Was the follow up of subjects long enough?	7. Do you believe the results?	8. Can the results be applied to the local population?	9. Do the results of this study fit with other available evidence?
	Benjumea-Bedoya, et al. 2019	Retrospective Cohort	9/11	Yes	Yes	Yes	Yes	can't tell	can't tell	Yes	Yes	yes	yes	Yes
	Bennet, et al. 2014	Retrospective Cohort	10/11	Yes	Yes	Yes	yes	Yes	Yes	yes	No	yes	yes	yes
	Bishara, et al. 2015	Retrospective Cohort	11/11	Yes	Yes	Yes	Yes	Yes	yes	Yes	Yes	Yes	Yes	Yes
	Brassard, et al. 2006	Retrospective Cohort	11/11	Yes	Yes	Yes	Yes	Yes	Yes	Yes	Yes	Yes	Yes	Yes
	Carter, et al. 2017	Retrospective Cohort	8/11	Yes	Yes	Yes	Yes	No	No	Yes	No	Yes	Yes	Yes
	Chang, et al. 2013	Retrospective Cohort	10/11	Yes	Yes	Can't tell	Yes	Yes	Yes	Yes	Yes	Yes	Yes	Yes
	Dobler, Marks. 2012	Retrospective Cohort	10/11	Yes	Yes	Can't tell	Yes	yes	yes	yes	yes	yes	yes	yes
	Duchen, et al. 2017	Retrospective Cohort	10/11	Yes	Yes	Yes	Yes	Yes	Can't tell	Yes	Yes	Yes	Yes	Yes
	Elliot, et al. 2018	Retrospective Cohort	8/11	Yes	Yes	Yes	Can't tell	no	no	yes	Yes	yes	yes	yes
	Essadek, et al. 2018	Retrospective Cohort	7/11	Yes	Yes	Can't tell	Can't tell	Yes	No	yes	can't tell	yes	yes	yes
	Gacek, et al. 2013	Retrospective Cohort	6/11	Yes	Yes	can't tell	Can't tell	Yes	no	No	can't tell	yes	yes	yes
	Goswamin, et al. 2012	Prospective Cohort	11/11	Yes	Yes	yes	Yes	Yes	Yes	yes	Yes	yes	yes	yes
	Haley, et al. 2008	Retrospective Cohort	10/11	Yes	Yes	Can't tell	Yes	Yes	Yes	Yes	Yes	Yes	Yes	Yes
	Harstad, et al. 2010	Prospective Cohort	11/11	Yes	Yes	Yes	Yes	Yes	Yes	Yes	Yes	Yes	Yes	Yes
	Kim, et al. 2019	Retrospective Cohort	10/11	Yes	can't tell	Yes	Yes	yes	yes	Yes	Yes	Yes	yes	Yes
	Lardizabal, et al. 2006	Retrospective Cohort	9/11	Yes	Yes	Can't tell	Yes	Yes	No	Yes	Yes	Yes	Yes	Yes
	Li, et al. 2010	Retrospective Cohort	11/11	Yes	Yes	Yes	Yes	Yes	Yes	Yes	Yes	Yes	Yes	Yes
	Lim, et al. 2016	Retrospective Cohort	10/11	Yes	Yes	Yes	yes	Yes	no	Yes	Yes	Yes	Yes	Yes
	Loutet, et al. 2018	Retrospective Cohort	11/11	Yes	Yes	Yes	Yes	Yes	Yes	Yes	Yes	Yes	Yes	Yes
	Nuzzo, et al. 2015	Retrospective Cohort	11/11	Yes	Yes	Yes	Yes	Yes	Yes	Yes	Yes	Yes	Yes	Yes
	O'Shea, et al. 2014	Prospective Cohort	8/11	Yes	Yes	Yes	Can't tell	No	No	Yes	Yes	Yes	Yes	Yes
	Olsson, et al. 2018	Retrospective Cohort	11/11	Yes	Yes	Yes	Yes	Yes	Yes	Yes	Yes	Yes	Yes	Yes

	Sawka, Brigham. 2019	Retrospective Cohort	6/11	Yes	Yes	Yes	Can't tell	can't tell	can't tell	yes	can't tell	yes	can't tell	yes
	Schein, et al. 2018	Prospective Cohort	10/11	Yes	Yes	Can't tell	Yes	Yes	Yes	Yes	Yes	Yes	Yes	Yes
	Shieh, et al. 2006	Prospective Cohort	10/11	Yes	Yes	Can't tell	Yes	Yes	Yes	Yes	Yes	Yes	Yes	Yes
	Sprujit, Erkens, et al. 2019	Retrospective Cohort	10/11	Yes	Yes	Yes	Can't tell	Yes	Yes	yes	yes	Yes	Yes	Yes
	Sprujit, Haile, et al. 2019	Prospective Cohort	10/11	Yes	Yes	Yes	Can't tell	Yes	Yes	yes	yes	Yes	yes	Yes
	Thee, et al. 2019	Prospecitve cohort	5/11	Yes	No	Yes	Can't tell	No	No	yes	can't tell	yes	no	yes
	Subedi, et al. 2015	Retrospective Cohort	8/11	Yes	Yes	Yes	Can't tell	Yes	No	yes	can't tell	Yes	Yes	Yes
	Trauer, Krause. 2011	Prospective Cohort	11/11	Yes	Yes	Yes	Yes	Yes	Yes	Yes	Yes	Yes	Yes	Yes
	Usdin, et al. 2017	Prospective Cohort	9/11	Yes	Yes	Yes	can't tell	Yes	No	Yes	Yes	Yes	Yes	Yes
	Villa, et al. 2019	Retrospective Cohort	10/11	Yes	Yes	Yes	Yes	Yes	No	Yes	Yes	Yes	Yes	Yes
	Walters, Sullivan. 2016	Retrospective Cohort	9/11	Yes	Yes	Yes	Can't tell	Yes	Yes	cant tell	Yes	Yes	Yes	Yes
Randomised Controlled-Trial	***Citation***	***Study Type***	***Score***	1. Did the trial address a clearly focused issue?	2. Was the assignment of patients to treatments randomised?	3. Were all of the patients who entered the trial properly accounted for at its conclusion?	4. were patients, health workers and study personnel 'blind' to treatment?	5. were the groups similar at the start of the trial?	6. Aside from the experimental intervention, were the groups treated equally?	7.Can the results be applied to the local population, or in your context?	8. Were all clinically important outcomes considered?	9. Are the benefits worth the harms and costs?		
	Hirsch-Moverman, et al. 2010	RCT	7/9	Yes	Yes	Yes	Yes	No	Yes	Can't tell	Yes	Yes		
	Jimenez-Fuentes, et al. 2013	RCT	8/9	Yes	Yes	Yes	Can't tell	Yes	Yes	Yes	Yes	Yes		

Appendix 5. Preferred Reporting Items for Systematic Reviews and Meta-Analyses (PRISMA) checklist.

Section/topic	#	Checklist item	Reported on page #
**TITLE**			
Title	1	Identify the report as a systematic review, meta-analysis, or both.	1
**ABSTRACT**			
Structured summary	2	Provide a structured summary including, as applicable: background; objectives; data sources; study eligibility criteria, participants, and interventions; study appraisal and synthesis methods; results; limitations; conclusions and implications of key findings; systematic review registration number.	1
**INTRODUCTION**			
Rationale	3	Describe the rationale for the review in the context of what is already known.	2-3
Objectives	4	Provide an explicit statement of questions being addressed with reference to participants, interventions, comparisons, outcomes, and study design (PICOS).	2-3
**METHODS**			
Protocol and registration	5	Indicate if a review protocol exists, if and where it can be accessed (e.g., Web address), and, if available, provide registration information including registration number.	3
Eligibility criteria	6	Specify study characteristics (e.g., PICOS, length of follow-up) and report characteristics (e.g., years considered, language, publication status) used as criteria for eligibility, giving rationale.	3-4
Information sources	7	Describe all information sources (e.g., databases with dates of coverage, contact with study authors to identify additional studies) in the search and date last searched.	3
Search	8	Present full electronic search strategy for at least one database, including any limits used, such that it could be repeated.	Appendix 1
Study selection	9	State the process for selecting studies (i.e., screening, eligibility, included in systematic review, and, if applicable, included in the meta-analysis).	3
Data collection process	10	Describe method of data extraction from reports (e.g., piloted forms, independently, in duplicate) and any processes for obtaining and confirming data from investigators.	3-4
Data items	11	List and define all variables for which data were sought (e.g., PICOS, funding sources) and any assumptions and simplifications made.	4

Risk of bias in individual studies	12	Describe methods used for assessing risk of bias of individual studies (including specification of whether this was done at the study or outcome level), and how this information is to be used in any data synthesis.	4
Summary measures	13	State the principal summary measures (e.g., risk ratio, difference in means).	4
Synthesis of results	14	Describe the methods of handling data and combining results of studies, if done, including measures of consistency (e.g., I^2) for each meta-analysis.	4
Section/topic	#	Checklist item	Reported on page #
Risk of bias across studies	15	Specify any assessment of risk of bias that may affect the cumulative evidence (e.g., publication bias, selective reporting within studies).	4
Additional analyses	16	Describe methods of additional analyses (e.g., sensitivity or subgroup analyses, meta-regression), if done, indicating which were pre-specified.	4
**RESULTS**			
Study selection	17	Give numbers of studies screened, assessed for eligibility, and included in the review, with reasons for exclusions at each stage, ideally with a flow diagram.	Figure 1; 5
Study characteristics	18	For each study, present characteristics for which data were extracted (e.g., study size, PICOS, follow-up period) and provide the citations.	Appendix 2.
Risk of bias within studies	19	Present data on risk of bias of each study and, if available, any outcome level assessment (see item 12).	Appendix 4.
Results of individual studies	20	For all outcomes considered (benefits or harms), present, for each study: (a) simple summary data for each intervention group (b) effect estimates and confidence intervals, ideally with a forest plot.	5-9
Synthesis of results	21	Present results of each meta-analysis done, including confidence intervals and measures of consistency.	5-9
Risk of bias across studies	22	Present results of any assessment of risk of bias across studies (see Item 15).	5-9
Additional analysis	23	Give results of additional analyses, if done (e.g., sensitivity or subgroup analyses, meta-regression [see Item 16]).	8
**DISCUSSION**			
Summary of evidence	24	Summarize the main findings including the strength of evidence for each main outcome; consider their relevance to key groups (e.g., healthcare providers, users, and policy makers).	9
Limitations	25	Discuss limitations at study and outcome level (e.g., risk of bias), and at review-level (e.g., incomplete retrieval of identified research, reporting bias).	10

Conclusions	26	Provide a general interpretation of the results in the context of other evidence, and implications for future research.	9-10
**FUNDING**			
Funding	27	Describe sources of funding for the systematic review and other support (e.g., supply of data); role of funders for the systematic review.	10
